# Negotiating uncertainty and responsibility after a vestibular schwannoma diagnosis: a qualitative study of meaning-making and surgical decision-making

**DOI:** 10.1186/s40359-026-05506-1

**Published:** 2026-09-05

**Authors:** Mareike Rutenkröger, Nora Nieke, Lisa Brandes, Isabelle Scholl

**Affiliations:** Department of Medical Psychology, University Medical Center Hamburg-Eppendorf, Martinistraße 52, 20246 Hamburg, Germany

**Keywords:** Vestibular schwannoma, Patient preferences, Shared decision-making, Quality of life, Acoustic neuroma

## Abstract

**Background:**

A diagnosis of vestibular schwannoma (VS) confronts patients with complex treatment decisions under conditions of uncertainty. While previous research has focused on treatment outcomes, symptom trajectories, and predictors of treatment choice, less is known about how patients psychologically process the diagnosis and construct meaning before surgery. This study explored how patients experience uncertainty, regain agency, and negotiate responsibility during the preoperative period.

**Methods:**

We conducted semi-structured interviews with twelve patients who underwent surgical resection of a VS within the previous five years. Interviews explored emotional, cognitive, and relational experiences following diagnosis and during treatment decision-making. Data were analyzed using qualitative content analysis.

**Results:**

Four interconnected themes described a process of reconstructing certainty and agency after diagnosis: (1) Diagnosis as biographical disruption, as patients experienced VS as a sudden event challenging their sense of normality, identity, and future expectations; (2) Meaning-making as an attempt to regain control, involving information-seeking, peer exchange, and reinterpretation of previous experiences; (3) Healthcare interactions as regulators of uncertainty, with communication quality, empathy, and consistency shaping confidence and emotional stability; and (4) Surgical decision-making as emotional, relational, and moral work, requiring patients to negotiate fear, trust, responsibility, and the burden of irreversible choices.

**Conclusion:**

The preoperative experience of patients with VS extends beyond symptom burden and treatment selection. Patients engage in complex processes of meaning-making and uncertainty management before deciding on treatment. Patient-centered care should integrate clear information with empathetic communication and support patients in navigating the emotional and moral dimensions of decision-making.

**Supplementary Information:**

The online version contains supplementary material available at https://doi.org/10.1186/s40359-026-05506-1.

## Introduction

Vestibular schwannomas (VS) are benign tumors that develop in the cerebellopontine angle and can affect hearing, balance, and facial nerve function. Patients diagnosed with VS face complex treatment decisions, as there are several options available, including watchful waiting, microsurgical resection, radiosurgery, and fractionated radiotherapy [1, 2]. Each option has different risks and benefits, so the decision-making process is highly individualized and challenging [3, 4]. Therefore, VS represents a context in which patients face complex decisions under uncertainty, making it a valuable case for understanding how individuals negotiate responsibility and agency in healthcare.

Previous research indicates that VS treatment decision-making is often clinician-driven with limited patient involvement. For example, Müller et al. [1] found that most patients in Germany were informed of only one treatment option, typically surgery, and that information about potential side effects was often inadequate. Moshtagi et al. [5] found that patients often sought multiple opinions, reflecting the complexity of decision-making and the need for shared decision-making (SDM) approaches. Evidence from broader neuro-oncology contexts suggests that patients generally prefer a collaborative or active role in decision-making and value outcomes such as quality of life, functional independence, and long-term well-being, rather than survival alone [6].

Graham et al. [2] demonstrated that involving VS patients in SDM significantly reduces decisional conflict; patients reported lower uncertainty when they perceived a high level of engagement in the decision-making process. Interventions designed to support SDM in neuro-oncology, such as decision aids, structured communication tools, and clinician training, have been shown to improve patients’ understanding of their condition and satisfaction with the decision-making process, although their implementation remains limited [7, 8]. Notably, analyses of neurosurgical consultations suggest that decision-making often remains largely clinician-guided, with insufficient attention to patients’ everyday life priorities and subjective concerns [9].

Most VS management research has relied on quantitative methods, including surveys, cohort studies, and predictive modeling, to examine treatment choices and outcomes [4, 5, 10, 11]. These studies have primarily focused on clinical predictors such as tumor size, Koos grade, age, and symptom progression as determinants of active treatment [4, 10]. Survey-based research further highlights the influence of physician recommendations, perceived invasiveness of treatment, and anticipated recovery on treatment selection [5, 12]. While these findings provide valuable insights into decision outcomes, they offer limited understanding of how patients experience the decision-making process emotionally and cognitively.

Although some studies have addressed decision-making processes and SDM in VS and neuro-oncology [1, 2], the preoperative emotional experiences of patients remain underexplored. Research on patients awaiting surgery across medical disciplines indicates that the preoperative period is characterized by heightened uncertainty, strong informational needs, desire for control, and sensitivity to the quality of communication with healthcare professionals [13]. Qualitative studies in VS and related conditions further demonstrate that anxiety, personal beliefs, prior experiences, and anticipated impacts on daily functioning shape how patients perceive treatment options and risks [14, 15]. Hearing and balance preservation, reduction of tinnitus, maintenance of energy, and overall quality of life are frequently reported as central patient priorities, underscoring the importance of integrating subjective experiences into clinical decision-making [6, 15].

Understanding patients’ emotional responses and coping strategies during the preoperative phase is therefore crucial for providing patient-centered care. This qualitative study focused exclusively on patients who ultimately underwent microsurgical resection for VS. By exploring how these patients processed their diagnosis, navigated uncertainty, and prepared for surgery, this study aimed to contribute to a deeper understanding of preoperative decision-making and to inform improvements in counseling practices, shared decision-making, and alignment of treatment strategies with individual patient goals. The primary aim was to explore a broad range of topics related to (1) experiences of the diagnostic process, (2) emotional and cognitive responses, and (3) interactions with healthcare professionals and social support networks, ensuring a comprehensive understanding of participants’ preoperative experiences.

## Methods

### Study design

A cross-sectional qualitative study was conducted using semi-structured videocall interviews. Reporting was guided by the Consolidated Criteria for Reporting Qualitative Research (COREQ) [16], see Suppl. I.

### Recruitment

Recruitment was facilitated through the German patient advocacy group *Vereinigung Akustikus Neurinom e.V.* (VAN). NN presented the study objectives at a national group meeting, and a study flyer was distributed across VAN’s regional groups starting on January 26, 2024. Participants were invited to take part via email. Initially, 18 potential participants had expressed interest, and 12 of them fulfilled the eligibility criteria, including: age > 18 years, German as native language, surgical removal of the tumor within the last 5 years, and access to a device with an internet connection for participation. These criteria were verified during an initial telephone screening.

### Procedures and ethics

The study received approval from the local Ethics Institutional Review Board (Lokale Psychologische Ethikkommission at UKE (LPEK), #LPEK-0694) and was conducted in accordance with the Declaration of Helsinki and all relevant guidelines and regulations. Written informed consent was obtained from all interested participants prior to enrollment. Participants were contacted via email to schedule the semi-structured interviews, which were conducted by NN (Master’s student in Psychology, trained by senior researcher MR) between February 19, 2024, and March 30, 2024. All videocall interviews were recorded using a digital tape recorder, with the average interview lasting 54 min. Only NN and the participant were present during the interview. The recordings were transcribed verbatim by NN for further analysis. Demographic and clinical data were collected through a questionnaire. Preoperative symptoms, including hearing impairment and acute hearing loss, were assessed by self-report and reflected participants’ own descriptions; no audiometric criteria were applied to define or verify these variables.

### Interview domains

The semi-structured interview guide (see Suppl. II) was developed as part of a broader study exploring patients’ experiences across the treatment trajectory of VS, including pre- and postoperative phases as well as self-management strategies. In the present analysis, the focus was on open-ended questions that elicited participants’ experiences during the preoperative phase, including their emotional, cognitive, and social responses following the diagnosis of VS. The preliminary guide was reviewed and refined with input from two VAN advocates, resulting in a modified final version with improved clarity, comprehensibility, and relevance. No repeat interviews were carried out and no field notes were made during the interviews.

### Data analysis

Descriptive data were analyzed using SPSS software. The qualitative data analysis involved coding the full text of twelve transcribed interviews using MAXQDA (Version 2024). Following Kuckartz [17], meaningful sections were used as units of coding, with a meaningful phrase as the minimum and a multi-phrase text segment as the maximum unit. Multiple codings were applied when necessary. The coding system was organized hierarchically and included two levels, encompassing four main categories and twelve subcategories specifically reflecting preoperative experiences (see Supp. III). The four initial deductive categories were derived from the predefined domains of the semi-structured interview guide rather than from a separate theoretical model: (1) experiences of receiving the diagnosis, (2) emotional and cognitive responses to the diagnosis, (3) decision-making processes regarding surgery, and (4) information-seeking and exchange with healthcare professionals and peers. These categories provided an initial organizational framework. To remain open to experiences not captured by this structure, NN subsequently performed inductive categorization across the full dataset, allowing categories and subcategories to be refined and expanded based on participants’ accounts. The preliminary coding system was reviewed and refined with MR and SW. NN then coded the full dataset using the final coding system, and LB recoded a subset of transcripts for validation. Conflicts were resolved through consensus discussion with MR. Data saturation was reached when no new relevant themes emerged (inductive thematic saturation) [18]. Transcripts and analyses were not returned to participants.

## Results

### Sample characteristics

The sample consisted of *n* = 12 participants aged 27 to 70 years (M = 49.58; SD = 11.88), seven of whom identified as female. Tumor size was most commonly classified as Koos grade 2 (50%), and the retrosigmoid approach was used in 83.3% of cases. Full demographic and clinical characteristics, including self-reported preoperative symptoms, are presented in Table 1.

**Table 1:** Sample characteristics

Demographic and clinical variables	Participants(N=12)
	n(%)
Education 10 years of schooling>11 years of schooling	3 (25)9 (75)
Occupation Employed Retired	10 (83.3)2 (16.7)
Time since diagnosis (years)2 3 4 >5	1 (8.3)3 (25)5 (41.7)3 (25)
Time since treatment (years)0-1 1-2 2-3 3-4 4-5	1 (8.3)2 (16.7)6 (50)1 (8.3)2 (16.7)
Tumor size (Koos)1234Not reported	1 (8.3)6 (50)2 (16.7)2 (16.7)1 (8.3)
Surgery Treatment Access Retrosigmoidal Transtemporal Not reported	10 (83.3)1 (8.3)1 (8.3)
Self-reported preoperative symptoms	n(%)
Acute hearing loss Dizziness Headache Hearing impairment Nausea Numbness of the lip Sinusitis Tinnitus	6 (50).05 (41.7)2 (16.7)6 (50.0)1 (8.3)1 (8.3)1 (8.3)8 (66.7)

### Themes

**Fig. 1:**
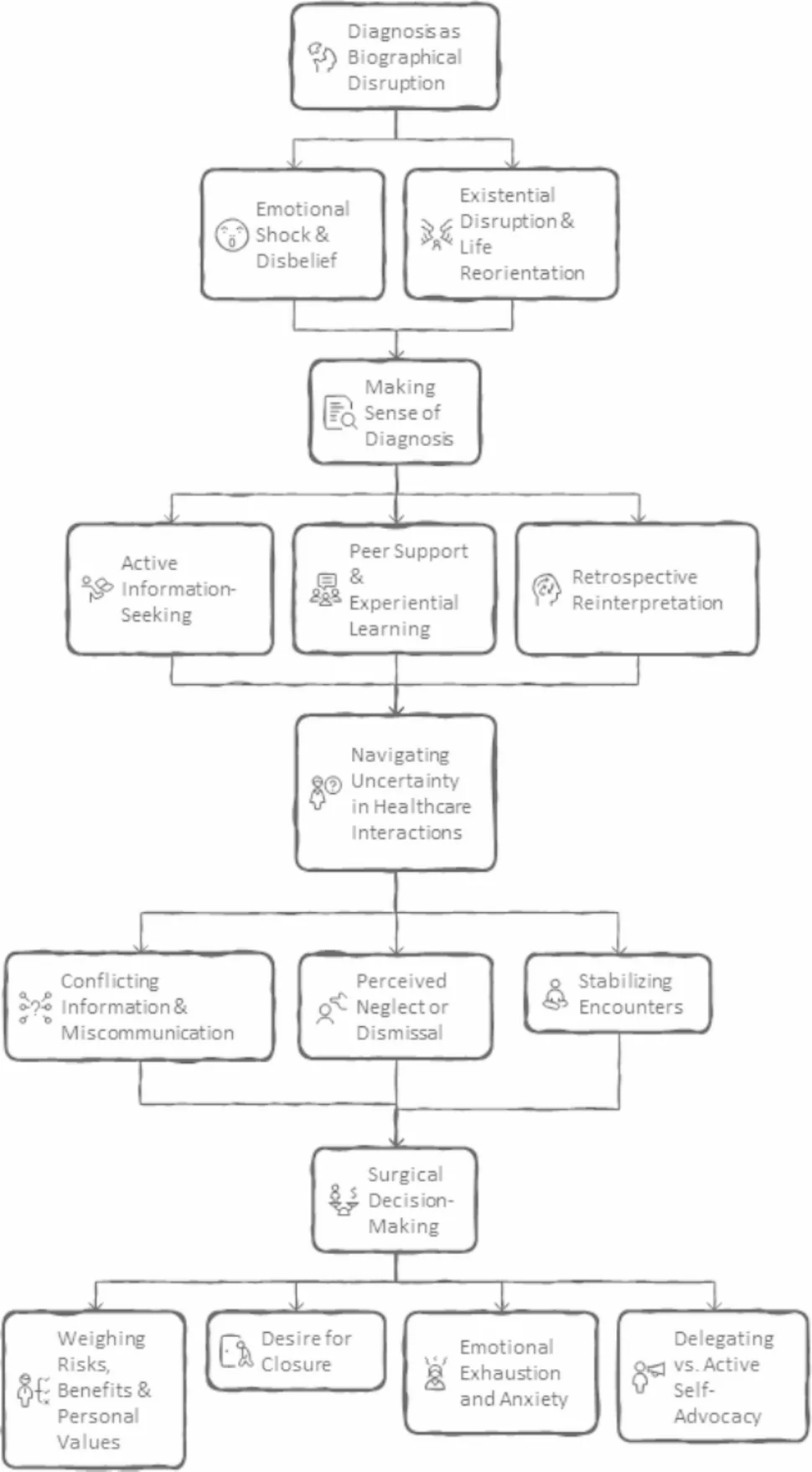
Emerged Themes and Subthemes representing preoperative experiences of decision-making in VS patients

Participants’ narratives indicated that a dynamic process of disruption, sense-making, uncertainty management, and decision-making characterized the preoperative period following VS diagnosis. Rather than focusing primarily on symptoms, participants described how the diagnosis affected their identity, understanding of their situation, interactions with healthcare professionals, and ability to make treatment decisions. Four interconnected themes and twelve subthemes were identified (Fig. 1). Illustrative quotations are provided in Table 2.

**Table 2:** Themes, Subthemes, and Illustrative Quotes

Theme	Subtheme	Participant ID & Example Quote (translated to English)
1. Diagnosis as a Biographical Disruption	1a. Emotional shock & disbelief	ID-1: “He was just as stunned as I was.”; ID-16: “It was kind of like a feeling of heaviness and shock.”; ID-8: “I didn’t really know what had happened to me. I felt like I’d been run over by a truck.”
	1b. Existential disruption & life reorientation	ID-10: “It was, of course, a time marked by great fear, great uncertainty, and very, very deep concerns about how life would go on after this surgery. Yes. But it was also a time when I received a lot of support, both personally and professionally. Not just negative experiences, but also a few positive ones.’”; ID-11: “I just asked myself, “What needs to change in my life?” That was a wake-up call for me. That’s often how it is when you get a diagnosis and ask yourself, “What’s going wrong?” Many people then start to rethink their lives. And I had similar thoughts along those lines.”
2. Making Sense of the Diagnosis Through Information and Meaning-Making	2a. Active information-seeking	ID-15: “And then I desperately tried to find out more: ‘Yes, what do I do now?’”; ID-10: “Of course, I first did some research on the Internet. I also visited the websites of some self-help groups.”; ID-1: “Then I looked into it a little bit… there aren’t too many studies… I had no symptoms and it was medium-sized.”; ID-2: “And the third thing was, of course, researching on the internet, obviously.”
	2b. Peer support & experiential learning	ID-9: “Then I contacted the VAN, which I found on the Internet… received good support.”; ID-16: “Then I joined the support group… where I met other people who had undergone radiation treatment.”; ID-10: “I spoke to people there who were, of course, already familiar with the disease.”
	2c. Retrospective reinterpretation	ID-9: “Then, when I knew that… what effects, what symptoms… some things in my life became clear to me.”; ID-7: “Due to the change of surgeon and a different type of consultation, I realized that this could actually be something drastic.”
3. Navigating Uncertainty in Interactions with Healthcare Professionals	3a. Conflicting information & miscommunication	ID-15: “First, no diagnosis: ‘There’s nothing wrong. Relax. It’s just stress-related.’ Then, the second time: ‘Yes, there’s a tumor… you need to go to the university hospital.’”; ID-16: “Then he went out and came back five minutes later: ‘Yes, you have a tumor. We can’t treat it here.’”
	3b. Perceived neglect or dismissal	ID-15: “I wasn’t entirely happy with the whole situation, including how the diagnosis was communicated.”; ID-15: “I often felt that I wasn’t really understood… they couldn’t put themselves in my shoes.”
	3c. Stabilizing encounters	ID-1: “Luckily, I knew about this… I was actually grateful.”; ID-8: “Now things are moving forward. Now you’ve found someone who really wants to help you… I’m incredibly grateful.”; ID-7: “Well, you can grow old with it… I didn’t really think about it that much anymore.”
4. Surgical decision-making as emotional, relational, and moral work	4a. Weighing risks, benefits & personal values	ID-10: “But radiation would have involved considerable risks. I changed my mind dozens of times during that period. First, I wanted to have radiation, then I wanted to have surgery. Then I went back and forth a few more times. In the end, I obviously had the surgery.”; ID-11: “And then he said it was too big because it expands after radiation treatment and then I would suffer too many brain failures… so I decided to have the operation.”; ID-1: “I didn’t want this thing to somehow hit the cerebellum… it was important to me that it happened beforehand.”;
	4b. Desire for closure	ID-1: “And I just wanted to get it sorted once and for all. Ideally, brain surgery rather than radiation therapy, otherwise in ten years I’ll be back at square one, wondering, ‘What am I going to do with this thing now?’”; ID-12: “I want to put this issue behind me.”; ID-11: “Okay, then I would have been rid of it too… the tumor.”; ID-16: “So I just made the decision: I’ll do it once and then it’ll be gone.”
	4c. Emotional exhaustion and anxiety during decision-making	ID-1: “The pre-phase, which lasted maybe three months, was of course the most difficult time… I found it much more emotionally stressful than the time before.”; ID-10: “It was a time marked by great fear, great uncertainty, and very, very great concerns about how life would continue after this operation.”; ID-15: “Yes, it was a very stressful time. There you are, with vestibular schwannoma, and what do you do with it now?”
	4d. Delegating vs. active self-advocacy	ID-13: “I thought: ‘Okay, this isn’t my area of expertise… I’ll do what the surgeons tell me to do.’”; ID-4: “After careful consideration… Prof. XY said… then I decided to go ahead with it.”; ID-10: “I then selected a few clinics that have good surgeons for this [VS]. I went there and spoke with them, listened to what they had to say on the subject.”

### Theme 1: Diagnosis as a Biographical Disruption

For most participants, receiving the diagnosis was a profound turning point accompanied by shock and disorientation. The realization of having a brain tumor challenged their sense of normalcy, established life narratives, and future expectations. Everyday routines and personal plans were called into question, and participants described a clear rupture between life “before” and “after” the diagnosis. Figure 1 summarizes the corresponding subthemes, with illustrative quotations provided in Table 2.

### Theme 2: Making Sense of the Diagnosis Through Information and Meaning-Making

After the initial shock, participants actively sought to understand their diagnosis and regain a sense of control. They described extensive information-seeking through internet research, consultations with healthcare professionals, and exchanges with other affected individuals. Participants also retrospectively reinterpreted previous bodily experiences in light of the diagnosis, integrating them into a more coherent illness narrative. Peer experiences provided additional reference points for contextualizing their situation and anticipating future developments (Fig. 1; Table 2).

### Theme 3: Navigating Uncertainty in Interactions with Healthcare Professionals

Interactions with healthcare professionals emerged as a central context in which uncertainty was either mitigated or intensified. Conflicting information, divergent recommendations, and communication perceived as rushed, vague, or dismissive contributed to confusion and insecurity. Conversely, clear explanations and empathetic engagement were described as stabilizing. Participants emphasized their dependence on professional guidance and described feeling particularly burdened when such guidance was fragmented or absent (Fig. 1; Table 2).

### Theme 4: Surgical Decision-Making as Emotional, Relational, and Moral Work

Participants described the decision to undergo surgery as extending beyond a rational evaluation of risks and benefits. Fear, responsibility, trust, and personal values shaped how they weighed treatment options and the possibility of making the “wrong” choice. Some reduced this burden by trusting specialist expertise, whereas others sought additional information or second opinions to maintain a sense of agency. Surgery was also frequently associated with a desire for closure and an end to prolonged uncertainty (Fig. 1; Table 2).

## Discussion

In this study, we explored how patients with VS experience the period between diagnosis and surgical treatment. Our findings suggest that the preoperative phase is not primarily characterized by symptom burden or the technical evaluation of treatment options, but by a complex psychological process of reconstructing certainty, meaning, and agency after a life-disrupting diagnosis. Across four interconnected themes, participants described how they first experienced diagnosis as a biographical disruption, subsequently attempted to make sense of their condition, relied on healthcare interactions to navigate uncertainty, and ultimately engaged in a decision-making process that involved emotional, relational, and moral considerations.

Rather than representing separate experiences, these themes describe a dynamic process through which patients attempted to regain control over an uncertain and potentially threatening future. This extends previous quantitative research on VS, which has predominantly focused on symptom trajectories, functional outcomes, treatment risks, and predictors of treatment selection [3–5, 10, 12]. While these outcomes remain clinically important, our findings highlight that the meaning patients assign to diagnosis and treatment decisions is shaped by processes that occur before clinical outcomes can even emerge.

Consistent with the concept of illness as biographical disruption [19], participants experienced the diagnosis of VS as a sudden interruption of their expected life trajectory (Theme 1). The diagnosis created a clear distinction between a “before” and “after,” challenging assumptions about health, normality, and future plans. Related qualitative research in patients with VS has likewise described substantial emotional responses and changes in personal priorities surrounding diagnosis and treatment decision-making [14, 15]. However, our findings extend previous work by demonstrating that, even in the context of a predominantly benign tumor, the diagnosis itself may represent a profound psychological event. Thus, the impact of VS should not be understood solely in relation to neurological symptoms or functional impairment, but also in relation to the disruption of personal narratives and identity.

Following this disruption, participants engaged in active meaning-making processes to restore coherence and regain a sense of control (Theme 2). Information-seeking, discussions with peers, and retrospective reinterpretation of previous symptoms served not only to acquire medical knowledge but also to integrate the diagnosis into an understandable personal narrative. Previous research has shown that patients with VS actively seek information to understand their condition and treatment options [1, 11]. Similarly, neuro-oncology research emphasizes that patients differ in their informational needs and benefit from individualized, comprehensible communication aligned with their values and coping capacities [6]. Our findings extend this literature by suggesting that information provision alone may be insufficient. Patients were not simply searching for facts; they were attempting to construct meaning and regain psychological stability in response to an unexpected threat. These processes may remain invisible in quantitative assessments of information needs or decisional conflict.

Healthcare interactions played a central role in determining whether uncertainty was experienced as manageable or overwhelming (Theme 3). Participants described that communication characterized by clarity, consistency, and empathy provided reassurance and helped them regain confidence, whereas fragmented information or emotionally distant interactions intensified insecurity. These findings complement previous research showing that patients with VS may experience limitations in information provision regarding treatment options, contributing to decisional conflict and dissatisfaction [1, 5]. While SDM has been demonstrated to reduce decisional conflict and support patient involvement [2], our findings suggest that SDM should not be conceptualized solely as the transfer of information or the presentation of treatment alternatives. Rather, effective decision-making requires a relational foundation in which patients feel understood, supported, and emotionally prepared to engage with uncertainty. This aligns with qualitative research in neurosurgical contexts demonstrating that treatment discussions may remain predominantly clinician-guided and insufficiently responsive to patients’ individual priorities and everyday concerns [6].

The most distinctive contribution of our study concerns the nature of surgical decision-making itself (Theme 4). Participants did not describe surgery as a purely rational comparison of risks and benefits but as a process involving fear, responsibility, trust, and moral considerations. Previous research on VS treatment decisions has primarily identified clinical factors associated with treatment choice, including tumor characteristics, age, and symptom progression [3, 4, 10]. Our findings suggest that this perspective captures only part of the decision-making process. Patients also had to negotiate questions of responsibility: How much uncertainty should they tolerate? Should they rely on expert recommendations or actively challenge them? How can they be confident that they have made the “*right*” decision?

This process can be understood as a form of moral work, in which patients attempt to reconcile competing values, responsibilities, and sources of knowledge. Some participants managed this burden by placing trust in specialist expertise and delegating parts of the decision-making process, whereas others sought additional information or opinions to maintain a sense of agency. Similar tensions between trust in clinicians and the desire for control have been described in qualitative research on VS treatment decision-making [14]. However, our findings highlight that decision-making in VS involves not only autonomy and preference expression but also the emotional burden of responsibility associated with irreversible choices.

Taken together, our findings suggest that the preoperative period in VS should be conceptualized as a critical phase of psychological adaptation rather than merely a pathway toward treatment selection. For skull base surgeons, several practical implications follow. Counseling should explicitly acknowledge uncertainty rather than focusing only on the technical risks and benefits of each option. Clinicians can reduce the perceived burden of individual responsibility by framing treatment selection as a shared clinical decision, clarifying where more than one option is medically reasonable, and exploring how patients’ values and everyday priorities shape the choice. Because information needs varied across participants, clinicians should assess how much and what type of information patients want, provide opportunities to revisit information over time, and help contextualize conflicting recommendations or information obtained from other sources. Empathic validation of fear and uncertainty, sufficient space for questions, and support in obtaining a second opinion when desired may further strengthen confidence in the decision-making process [20]. Decision support tools and counseling interventions tailored to VS may complement, rather than replace, these relational aspects of surgical counseling [8, 21].

Future research should investigate how these psychological and relational processes differ among patients choosing surgery, active surveillance, or other treatment approaches. Comparative studies including patients with different management strategies may further clarify how uncertainty, agency, and responsibility shape treatment decisions over time.

Strengths and limitations.

This study provides an in-depth exploration of the emotional, cognitive, and relational processes underlying the preoperative experiences of patients with VS. The qualitative design enabled investigation of subjective experiences that are difficult to capture using quantitative measures alone. The combined deductive-inductive qualitative content analysis provided a structured link to the predefined interview domains while allowing categories and subcategories to be refined and expanded from participants’ accounts.

Several limitations should be considered. First, participants were recruited through a patient advocacy organization, which may have introduced self-selection bias. Previous research comparing samples recruited through a patient-focused VS organization with a tertiary-care cohort has demonstrated systematic differences between these recruitment sources [22]. In addition, the sample was relatively homogeneous and highly educated, with 75% of participants reporting more than 11 years of schooling. The findings may therefore underrepresent perspectives of patients with lower educational attainment or those who are less engaged with patient organizations. A purposive sampling strategy aimed at greater variation in educational and clinical characteristics could strengthen future studies. Second, the initial deductive categories were aligned with the predefined interview domains. Although subsequent inductive analysis was used to refine and expand the coding system, this starting structure may have directed analytic attention toward these domains and constrained the identification of experiences outside them. Third, participants were interviewed up to five years after surgery, and retrospective accounts may have been influenced by subsequent experiences, memory processes, or post hoc meaning-making. However, these retrospective narratives also provide insight into how patients reconstruct and interpret major health-related experiences over time. Fourth, the study included only patients who underwent surgery and therefore does not capture perspectives of individuals who selected alternative management strategies, such as observation or radiotherapy. Finally, the small sample and its distribution across Koos grades did not permit robust comparisons of experiences between patients with Koos 1–2 and Koos 3–4 tumors. Future purposively sampled studies should examine whether the degree of clinical ambiguity associated with tumor size or stage systematically shapes uncertainty and decision-making. Future research should also include different treatment pathways to explore how management strategy influences experiences of uncertainty, agency, and decision-making.

## Conclusion

This study demonstrates that the preoperative experience of patients with vestibular schwannoma extends beyond clinical symptoms and treatment selection. Patients undergo a complex process of reconstructing meaning, managing uncertainty, and negotiating responsibility after a life-disrupting diagnosis. By highlighting the emotional and relational dimensions of surgical decision-making, our findings emphasize the need for patient-centered approaches that integrate medical information with empathetic communication and support for patients’ psychological adaptation processes.

## Supplementary Information

Below is the link to the electronic supplementary material.

Supplementary Material 1

Supplementary Material 2

Supplementary Material 3

## Data Availability

The datasets used and/or analyzed during the current study are available from the corresponding author on reasonable request.

## Abbreviations

VS: Vestibular Schwannoma
SDM: Shared Decision-Making
VAN: Vereinigung Akustikus Neurinom e.V
