# Supplementary material for: Negotiating uncertainty and responsibility after a vestibular schwannoma diagnosis: a qualitative study of meaning-making and surgical decision-making
Source: BMC Psychol. 2026 Sep 5;14:1289. doi: 10.1186/s40359-026-05506-1 (PMC13546501; doi:10.1186/s40359-026-05506-1)
Supplement: Supplementary file 2 [file 40359_2026_5506_MOESM2_ESM.docx]

**Supplemenatry Material III. Coding Tree.**

| Theme | Subtheme | Description |
| --- | --- | --- |
| 1. Diagnosis as a Biographical Disruption | 1a. Emotional shock & disbelief | Participants described the moment of diagnosis as a sudden emotional upheaval characterized by shock, disbelief and a sense of being overwhelmed. This was often accompanied by feelings of helplessness and physical distress. |
|  | 1b. Existential disruption & life reorientation | Beyond the initial shock, the diagnosis prompted participants to reflect more deeply on their lives, their future expectations and their previous assumptions about health and vulnerability, leading them to reassess their life trajectories. |
| 2. Making Sense of the Diagnosis Through Information and Meaning-Making | 2a. Active information-seeking | Participants sought information about their condition, both medical and experiential, primarily through online research and self-help resources, in order to regain a sense of control and better understand the implications of the diagnosis. |
|  | 2b. Peer support & experiential learning | Engaging with peers affected by the same condition can provide emotional reassurance and practical knowledge. This can often play a decisive role in treatment preferences and decision-making. |
|  | 2c. Retrospective reinterpretation | Following their diagnosis, participants reinterpreted their past bodily sensations, symptoms and experiences retrospectively, incorporating them into a newly constructed narrative of illness. |
| 3. Navigating Uncertainty in Interactions with Healthcare Professionals | 3a. Conflicting information & miscommunication | Participants reported experiencing contradictory medical advice and abrupt communication during the diagnostic process, which heightened uncertainty and undermined trust. |
|  | 3b. Perceived neglect or dismissal | Some participants felt that they had not been adequately acknowledged by healthcare professionals. They described encounters that were characterized by emotional distance, a lack of empathy and an insufficient validation of their concerns. |
|  | 3c. Stabilizing encounters | By contrast, supportive and reassuring interactions with healthcare professionals helped to reduce anxiety, foster trust and contribute to emotional stabilization and acceptance of the diagnosis. |
| 4. Surgical decision-making as emotional, relational, and moral work | 4a. Weighing risks, benefits & personal values | Treatment decisions required complex deliberations, weighing up medical risks and benefits against personal values, life priorities and fears of functional loss or disease progression. |
|  | 4b. Desire for closure | Many participants expressed a strong desire for decisiveness and finality. They viewed surgery as a means of eliminating ongoing uncertainty and of 'closing the chapter' on their illness. |
|  | 4c. Emotional exhaustion and anxiety during decision-making | The prolonged decision-making process was described as emotionally taxing, characterized by intense anxiety and fear for the future, as well as sustained psychological strain. |
|  | 4d. Delegating vs. active self-advocacy | The participants’ decision-making roles differed, ranging from delegating responsibility to medical experts, to actively seeking multiple opinions, to asserting autonomy in treatment choices. |
