# Supplementary material for: Negotiating uncertainty and responsibility after a vestibular schwannoma diagnosis: a qualitative study of meaning-making and surgical decision-making
Source: BMC Psychol. 2026 Sep 5;14:1289. doi: 10.1186/s40359-026-05506-1 (PMC13546501; doi:10.1186/s40359-026-05506-1)
Supplement: Supplementary file 3 [file 40359_2026_5506_MOESM3_ESM.docx]

Qualitative Interview Guide

*Qualitative interviews with patients with vestibular schwannoma as part of the study: “Experiences and Self-Management Strategies of Patients with Vestibular Schwannoma in Coping with Biopsychosocial Challenges During the Postoperative Phase”*

# Introduction

**Thanking the participant**
First of all, I would like to sincerely thank you for agreeing to participate in this interview.

**Introducing the interviewer**
My name is Nora Nieke, and I am a Master's student in Psychology at the University of Lübeck. As part of my Master's thesis, I am conducting a study together with the Psycho-Oncology Research Group at the University Medical Center Hamburg-Eppendorf on the experiences of people with vestibular schwannoma and how they cope with challenges following surgery.

**Informed consent**
Although you have already provided written informed consent, you may ask to stop the interview at any time and request that all data collected up to that point be deleted. During this interview, I will ask you some personal questions. If any question makes you feel uncomfortable, you may choose not to answer it.

**Data protection**
Our interview will be recorded using two audio-recording devices and subsequently transcribed to facilitate analysis of the information provided. All information you disclose during this interview will be treated confidentially. During transcription, the interview will be anonymized so that no conclusions can be drawn about your identity.

All audio recordings will be deleted three months after transcription has been completed. The transcripts will be destroyed ten years after completion of the study. Until then, only members of the Psycho-Oncology Research Group at the University Medical Center Hamburg-Eppendorf will have access to the data. Detailed information is provided in the participant information sheet and informed consent form.

**Duration**
The interview will take approximately 45–60 minutes.

**Questions**
Do you have any further questions regarding the content or procedure of the interview or any aspects related to data protection?

We will now begin the interview and start the audio recording.

[Switch on recording device.]

**Confirmation of consent after starting the recording**
I have now started the audio recording. Is that okay with you?

Then we will begin.

# Interview Introduction

In today's interview, I would like to ask you about your personal experiences with biological and psychosocial challenges following surgery for vestibular schwannoma. The interview will focus particularly on how you dealt with these challenges, the approaches you took, and the personal strategies you used to cope with them.

There are no right or wrong answers to these questions; I am interested in your individual perspectives and experiences. At the end of the interview, you will have the opportunity to raise any additional aspects that have not been covered by the questions. Before then, however, I would ask you to answer the questions as specifically and directly as possible.

# Main Interview

*Structured according to the domains of the biopsychosocial model.*

## Part 1: Experiences During the Preoperative Phase

First, I would like to talk with you about your experiences during the period between receiving your diagnosis and undergoing surgery. Please think back to that time.

**Main questions**

- How did you come to receive the diagnosis of vestibular schwannoma?
- How did you experience receiving the diagnosis? What thoughts and feelings did you have after receiving the diagnosis?
- Did you experience any symptoms before surgery? If so, which symptoms?

**Additional prompts**

- How did you obtain information about vestibular schwannoma and the available treatment options?
- What experiences did you have in your interactions with healthcare professionals after receiving the diagnosis and while considering treatment?
- How did the decision to undergo surgery come about? What was particularly important to you when making this decision?
- How would you describe your quality of life after receiving the diagnosis and before surgery?
- What is the first thing that comes to mind when you think about the period after receiving the diagnosis and before surgery? Which experiences from this period have remained particularly vivid in your memory?
- Were there particular aspects or situations during this period that you found especially helpful or particularly challenging?

## Part 2: Biological Changes During the Postoperative Phase

We will now look in turn at your personal experiences with physical, psychological, and social changes that may have occurred after surgery. First, we will focus exclusively on the physical domain.

**Main questions**

- Did you experience any physical changes after surgery? If so, which ones?
- What specific experiences did you have in relation to these physical changes?

**Additional prompts**

- Did you experience any physical symptoms after surgery that you had not experienced before, or did any existing symptoms worsen?
- Were there particular aspects or situations that you found especially challenging?

**Possible follow-up prompts**

- Tinnitus?
- Dizziness?
- Hearing problems?
- Postoperative headache?

**Main questions**

- How did you cope with the physical changes you have just described?
- What strategies or methods did you use to manage these changes?

**Additional prompts**

- What did you do to cope with the physical changes?
- Did you develop new strategies or adapt strategies you were already using?
- Can you give specific examples of strategies that helped you cope with these changes?
- How or where did you learn about these strategies?
- What helped you identify strategies that worked for you?

**Main questions**

- What experiences did you have when using these strategies?
- What would have helped you use these strategies more effectively?
- What challenges did you encounter when using these strategies?

**Additional prompts**

- What helped you implement the strategies you described?
- Did you receive any support in using these strategies?
- Did the strategies you used change over time? If so, how did they change?
- What did you adapt the strategies to, if applicable (e.g., immediately after surgery compared with several months or years later)?
- Were there any barriers or obstacles that made it difficult to implement certain strategies?
- What would have made it easier for you to use these strategies in your everyday life?
- What additional support for implementing these strategies would have been helpful to you?

## Part 3: Psychological Changes During the Postoperative Phase

Having discussed the physical domain in detail, I would now like to turn to psychological changes.

**Main questions**

- Did you experience any psychological changes after surgery? If so, which ones?
- What specific experiences did you have in relation to these psychological changes?

**Additional prompts**

- Did you experience any psychological symptoms after surgery that you had not experienced before, or did any existing symptoms worsen?
- Were there particular aspects or situations that you found especially challenging?

**Possible follow-up prompts**

- Depressive symptoms?
- Anxiety?
- Finding meaning?
- Personal growth or development?
- Spirituality?
- Existential changes?
- Fear of disease progression?
- Hopelessness?
- Confidence or hope?
- Cognitive impairments?
- Changes in your perception of yourself or your self-image?
- Self-efficacy?

**Main questions**

- How did you cope with the psychological changes you have just described?
- What strategies or methods did you use to manage these changes?

**Additional prompts**

- What did you do to cope with these changes?
- Did you develop new strategies or adapt strategies you were already using?
- Can you give specific examples of strategies that helped you cope with these changes?
- How or where did you learn about these strategies?
- What helped you identify strategies that worked for you?

**Main questions**

- What experiences did you have when using these strategies?
- What would have helped you use these strategies more effectively?
- What challenges did you encounter when using these strategies?

**Additional prompts**

- What helped you implement the strategies you described?
- Did you receive any support in using these strategies?
- Did the strategies you used change over time? If so, how did they change?
- What did you adapt the strategies to, if applicable (e.g., immediately after surgery compared with several years later)?
- Were there any barriers or obstacles that made it difficult to implement certain strategies?
- What would have made it easier for you to use these strategies in your everyday life?
- What additional support for implementing these strategies would have been helpful to you?

## Part 4: Social Changes During the Postoperative Phase

Finally, we will consider the last domain and focus on social changes. Social changes may include, for example, changes resulting from the condition in your personal, family, or professional situation, your leisure activities, your ability to continue working, or your relationships with family members.

**Main questions**

- Did you experience any social changes after surgery? If so, which ones?
- What specific experiences did you have in relation to these social changes?

**Additional prompts**

- How did your professional and social situation change after surgery?
- Were there particular aspects or situations that you found especially challenging?

**Possible follow-up prompts**

- Leisure activities/hobbies?
- Personal interests?
- Relationships?
- Partnership?
- Friendships?
- Work/professional life?
- Role within the family?

**Main questions**

- How did you cope with the social changes you have just described?
- What strategies or methods did you use to manage these changes?

**Additional prompts**

- What did you do to cope with these changes?
- Did you develop new strategies or adapt strategies you were already using?
- Can you give specific examples of strategies that helped you cope with these changes?
- How or where did you learn about these strategies?
- What helped you identify strategies that worked for you?

**Main questions**

- What experiences did you have when using these strategies?
- What would have helped you use these strategies more effectively?
- What challenges did you encounter when using these strategies?

**Additional prompts**

- What helped you implement the strategies you described?
- Did you receive any support in using these strategies?
- Did the strategies you used change over time? If so, how did they change?
- What did you adapt the strategies to, if applicable (e.g., immediately after surgery compared with several years later)?
- Were there any barriers or obstacles that made it difficult to implement certain strategies?
- What would have made it easier for you to use these strategies in your everyday life?
- What additional support for implementing these strategies would have been helpful to you?

## Part 5: General Understanding of Self-Management

As part of this study, we are investigating different self-management strategies. To do so, it is helpful for us to understand what the term “self-management” means to you personally.

**Main question**

- What do you understand by the term “self-management”?

**Additional prompts**

- What, for example, do you consider to be part of self-management? What kinds of behaviors or activities?
- What is your attitude toward self-management? What do you think about it?
- In your opinion, what are the advantages and disadvantages of self-management?

# Concluding Questions

- Is there anything else you would like to tell me that I may not have asked about so far?
- Thinking back over all the changes we have discussed today, which aspects are particularly important to you?
- Is there anything we have not yet discussed that you feel should definitely be included?

# Closing

**Ending the recording**
I will now stop the audio recording.

[Switch off recording device.]

**Participant questionnaire**
Finally, I would briefly like to remind you about the questionnaire I sent you. If you have not yet completed it, I would kindly ask you to do so.
